# Association of Race and Ethnicity With Glycemic Control and Hemoglobin A_1c_ Levels in Youth With Type 1 Diabetes

**DOI:** 10.1001/jamanetworkopen.2018.1851

**Published:** 2018-09-07

**Authors:** Anna R. Kahkoska, Christina M. Shay, Jamie Crandell, Dana Dabelea, Giuseppina Imperatore, Jean M. Lawrence, Angela D. Liese, Cate Pihoker, Beth A. Reboussin, Shivani Agarwal, Janet A. Tooze, Lynne E. Wagenknecht, Victor W. Zhong, Elizabeth J. Mayer-Davis

**Affiliations:** 1 Department of Nutrition, University of North Carolina at Chapel Hill; 2 American Heart Association, Dallas, Texas; 3 School of Nursing, University of North Carolina at Chapel Hill; 4 Department of Biostatistics, Gillings School of Global Public Health, University of North Carolina at Chapel Hill; 5 Department of Epidemiology, Colorado School of Public Health, Aurora; 6 Division of Diabetes Translation, Centers for Disease Control and Prevention, Atlanta, Georgia; 7 Department of Research & Evaluation, Kaiser Permanente Southern California, Pasadena; 8 Department of Epidemiology and Biostatistics, University of South Carolina, Columbia; 9 Department of Pediatrics, University of Washington, Seattle; 10 Department of Biostatistical Sciences, Wake Forest School of Medicine, Winston-Salem, North Carolina; 11 Department of Endocrinology, Diabetes, and Metabolism, Perelman School of Medicine, University of Pennsylvania, Philadelphia; 12 Department of Preventive Medicine, Northwestern University Feinberg School of Medicine, Chicago, Illinois; 13 Department of Medicine, University of North Carolina at Chapel Hill

## Abstract

**Importance:**

Health disparities in the clinical presentation and outcomes among youth with type 1 diabetes exist. Long-term glycemic control patterns in racially/ethnically diverse youth are not well described.

**Objectives:**

To model common trajectories of hemoglobin A_1c_ (HbA_1c_) among youth with type 1 diabetes and test how trajectory group membership varies by race/ethnicity.

**Design, Setting, and Participants:**

Longitudinal cohort study conducted in 5 US locations. The analysis included data from 1313 youths (aged <20 years) newly diagnosed in 2002 through 2005 with type 1 diabetes in the SEARCH for Diabetes in Youth study (mean [SD] age at diabetes onset, 8.9 [4.2] years) who had 3 or more HbA_1c_ study measures during 6.1 to 13.3 years of follow-up. Data were analyzed in 2017.

**Exposures:**

Self-reported race/ethnicity.

**Main Outcomes and Measures:**

Hemoglobin A_1c_ trajectories identified through group-based trajectory modeling over a mean (SD) of 9.0 (1.4) years of diabetes duration. Multinomial models studied the association of race/ethnicity with HbA_1c_ trajectory group membership, adjusting for demographic characteristics, clinical factors, and socioeconomic position.

**Results:**

The final study sample of 1313 patients was 49.3% female (647 patients) with mean (SD) age 9.7 (4.3) years and mean (SD) disease duration of 9.2 (6.3) months at baseline. The racial/ethnic composition was 77.0% non-Hispanic white (1011 patients), 10.7% Hispanic (140 patients), 9.8% non-Hispanic black (128 patients), and 2.6% other race/ethnicity (34 patients). Three HbA_1c_ trajectories were identified: group 1, low baseline and mild increases (50.7% [666 patients]); group 2, moderate baseline and moderate increases (41.7% [548 patients]); and group 3, moderate baseline and major increases (7.5% [99 patients]). Group 3 was composed of 47.5% nonwhite youths (47 patients). Non-Hispanic black youth had 7.98 higher unadjusted odds (95% CI, 4.42-14.38) than non-Hispanic white youth of being in the highest HbA_1c_ trajectory group relative to the lowest HbA_1c_ trajectory group; the association remained significant after full adjustment (adjusted odds ratio of non-Hispanic black race in group 3 vs group 1, 4.54; 95% CI, 2.08-9.89). Hispanic youth had 3.29 higher unadjusted odds (95% CI, 1.78-6.08) than non-Hispanic white youth of being in the highest HbA_1c_ trajectory group relative to the lowest HbA_1c_ trajectory group; the association remained significant after adjustment (adjusted odds ratio of Hispanic ethnicity in group 3 vs group 1, 2.24; 95% CI, 1.02-4.92). In stratified analyses, the adjusted odds of nonwhite membership in the highest HbA_1c_ trajectory remained significant among male patients and youth diagnosed at age 9 years or younger, but not female patients and youth who were older than 9 years when they were diagnosed (*P* for interaction = .04 [sex] and .02 [age at diagnosis]).

**Conclusions and Relevance:**

There are racial/ethnic differences in long-term glycemic control among youth with type 1 diabetes, particularly among nonwhite male patients and nonwhite youth diagnosed earlier in life.

## Key Points

**Question:**

Is there evidence for racial/ethnic health inequity with respect to longitudinal patterns of glycemic control among youth with type 1 diabetes?

**Findings:**

In a longitudinal cohort study of 1313 youths (aged <20 years) with type 1 diabetes, patients with black race or Hispanic ethnicity were at higher risk of being in the highest and most rapidly increasing hemoglobin A_1c_ trajectory group over 9 years after diabetes diagnosis when compared with non-Hispanic white patients. These associations persisted only among male patients and those with diagnosis at age 9 years or younger.

**Meaning:**

There is health inequity with regard to glycemic control, particularly among young nonwhite male patients and nonwhite youth diagnosed earlier in life.

## Teaser

This longitudinal cohort study compares trajectories of hemoglobin A_1c_ (HbA_1c_) increase, considered a measure of glycemic control, among patients younger than 20 years of age with type 1 diabetes according to their self-reported race/ethnicity using data from the SEARCH for Diabetes in Youth study.

## Introduction

Type 1 diabetes (T1D) treatment is centered around the improvement and maintenance of tight glycemic control, as assessed by levels of hemoglobin A_1c_ (HbA_1c_), to prevent acute and chronic diabetes-related complications.^1,2,3^ Glycemic control can vary considerably from diabetes onset through adolescence,^4,5,6^ where fluctuations are known to occur during puberty^3,4,7,8,9,10,11,12^ and during early adulthood. Poorer glycemic control during early adulthood or from childhood to young adulthood has been attributed to a lack of continuity in diabetes-related clinical care^4,11,12^ as well as changes in self-care as children and adolescents with T1D grow into adulthood.^9,10,13^ However, glycemic control in youth and young adults with T1D is critical, as a higher average HbA_1c_ level in this period of development is associated with impaired growth as well as diabetic complications.^14,15,16,17^

In cross-sectional studies of adolescents and young adults, glycemic control differs by racial and ethnic subgroups.^18^ African American, American Indian, Hispanic, and Asian or Pacific Islander youth with T1D are more likely to have higher HbA_1c_ levels compared with non-Hispanic white youth.^19^ In longitudinal studies, nonwhite youth with T1D have increased markers of poor prognosis at diagnosis and 3 years following diagnosis, including higher HbA_1c_ levels, more frequent diabetic ketoacidosis, and severe hypoglycemia.^20^ A constellation of sociodemographic factors related to race/ethnicity and glycemic control have been proposed, ranging from family dynamics, depressive symptoms, and quality of life^13,21,22,23,24,25^ to diabetes regimen.^26,27,28^ The role of socioeconomic position as a mediator of racial/ethnic associations remains controversial.^28,29,30,31^ Additionally, health care–specific factors such as disparities in health literacy, diabetes-related knowledge, or access to health care are known to contribute to pediatric health disparity but have not been well explored in T1D.^32,33^

Latent class trajectory modeling has been used to identify subgroups who share a similar trajectory of HbA_1c_ over time.^34^ Few studies have examined whether racial/ethnic disparities in glycemic control persist over time from childhood into young adulthood among individuals with T1D. Our objective was to first visualize major trajectories of glycemic control from childhood into young adulthood using all data from youth of all racial and ethnic groups and to then characterize specific associations between race/ethnicity and distinct longitudinal patterns of glycemic control. Our hypothesis was that non-Hispanic black and Hispanic youth would be more likely than non-Hispanic white youth to have unfavorable trajectory patterns representing poor glycemic control and that this association may be mediated by clinical factors such as diabetes regimen^26,27,28^ and by socioeconomic position.^29,30,31^

## Methods

### Study Population

**Figure 1.**
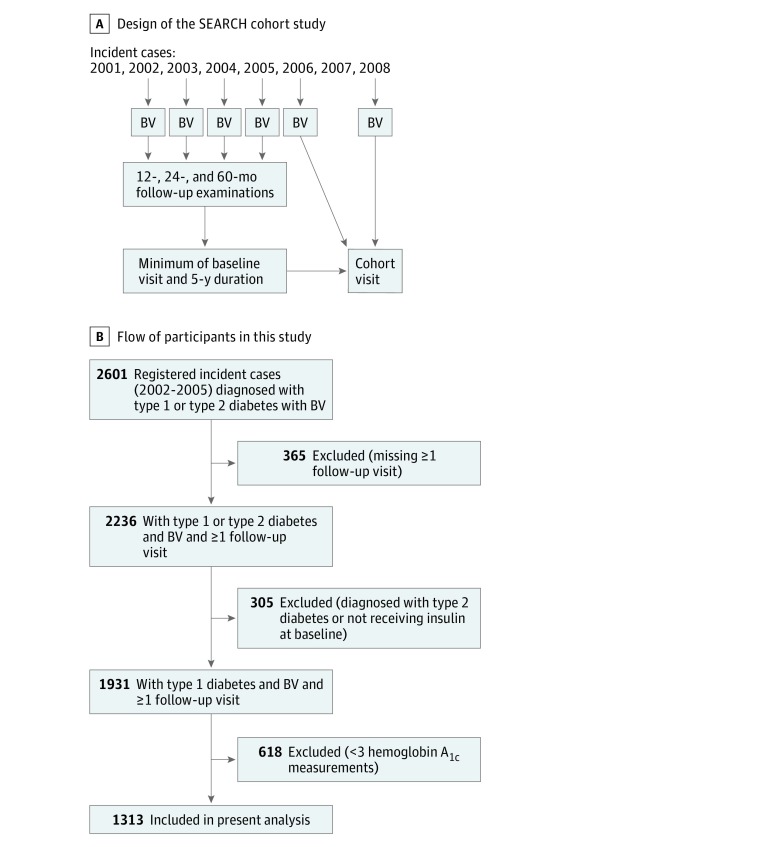
Study Design and Sample Recruitment A, Study design of the SEARCH cohort study. B, Flowchart depicting participants in this report, including reasons for exclusion. The final sample included 1313 youths with type 1 diabetes. BV indicates baseline visit.

The SEARCH for Diabetes in Youth study began in 2000 with an overarching objective to describe the incidence and prevalence of childhood diabetes among the 5 major racial and ethnic groups in the United States.^35^ Individuals with diabetes diagnosed before age 20 years were identified from a population-based incidence registry network at 5 US sites (South Carolina; Cincinnati, Ohio, and surrounding counties; Colorado with southwestern Native American sites; Seattle, Washington, and surrounding counties; and Kaiser Permanente, southern California).^36^ Patients were newly diagnosed with T1D in 2002 through 2005. Patients who could be contacted were asked to complete a short survey and recruited for a baseline visit. If they completed the first visit, they were asked to return for visits at 12, 24, and 60 months to measure risk factors for diabetes complications (Figure 1A). A subset of participants who were aged 10 years and older and had at least 5 years of diabetes duration were recruited for a follow-up cohort visit between 2012 and 2015. The subset of youth who were included in the SEARCH cohort visit were not significantly different from all other youth diagnosed between the years of 2002 and 2008 in terms of average age at diabetes onset, distribution of sex or race and ethnicity, or clinical measures.^14^

Inclusion criteria for these analyses consisted of youth diagnosed with T1D between 2002 and 2005. Type 1 diabetes was based on the clinical diagnosis made by a physician or other health care professional at onset and was collected from these health care professionals or abstracted from medical records. Youth with a clinical diagnosis of type 1a, type 1b, or type 1 diabetes were included. Youth who had fewer than 3 measures of HbA_1c_ from research visits during 6.1 to 13.3 years of follow-up were excluded (n = 618). Excluded individuals were not different with regard to HbA_1c_ measures using available data from the study baseline and the cohort visit. The final study sample included 1313 youths with T1D (Figure 1B). The study was approved by institutional review boards with jurisdiction; the parent, the participant, or both provided written consent or assent for all participants (consent of ≥1 parent or legal guardian was required for participants aged <18 years). The study followed the Strengthening the Reporting of Observational Studies in Epidemiology (STROBE) reporting guideline.

### Research Visits

Trained personnel administered questionnaires; measured height, weight, and blood pressure; and obtained blood samples. Body mass index was defined as weight (kilograms) divided by height (meters squared) and converted to a *z* score.^37^ A blood draw occurred after an 8-hour overnight fast, and medications, including short-acting insulin, were withheld the morning of the visit.

### Laboratory Measures

Blood samples were obtained under conditions of metabolic stability, defined as no episodes of diabetic ketoacidosis in the preceding month and the absence of fever and acute infections. They were processed locally and shipped within 24 hours to the central laboratory (Northwest Lipid Metabolism and Diabetes Research Laboratories). Hemoglobin A_1c_ was measured by a dedicated ion exchange high-performance liquid chromatography instrument (TOSOH Bioscience).

### Other Measures

Self-reported race and ethnicity were collected based on questions modeled after the 2000 US Census^38^ and categorized as non-Hispanic white, non-Hispanic black, Hispanic, and other (Asian, Native American, Pacific Islander, other, and unknown). Although the US Census accommodates reporting of multiple races, the SEARCH study did not have sufficient participant numbers to allow evaluation of separate categories of reported multiple-race groups^39^ and used the National Center for Health Statistics plurality approach, in which data from a study designed to address multiple-race reporting was used to determine which single-race category should be assigned for specific combinations of multiple races reported.^38^

Insulin regimen was based on mode of insulin delivery, classified as pumps, long-acting with rapid-acting insulin injections with 3 or more injections per day, and any other form of multiple daily injections. Insulin dose was reported as units per kilogram of body weight. Frequency of self-monitoring of blood glucose was self-reported and categorized as less than 1 time per day, 1 to 3 times per day, and 4 or more times per day. Health insurance type was classified as none, private, Medicaid, or other. Parental education was based on the highest educational level attained by either parent and classified as less than high school degree, high school graduate, some college through associate’s degree, and bachelor’s degree or more. Household structure was classified as 2 parent, single parent, or other. Receipt of diabetes care was based on reported number of visits with prespecified diabetes health care professionals, including pediatric endocrinologists, adult diabetologists, and nurse diabetes educators, in the previous 6 months and classified based on the distribution: 0 to 1 visit, 2 to 3 visits, 4 to 5 visits, and 6 or more visits. Receipt of nondiabetes care was based on reported number of visits with prespecified nondiabetes health care professionals (pediatrician, family practice physician, general practice physician, internist, nurse practitioner or physician assistant, traditional healer, dietician, optometrist or ophthalmologist, and psychiatrist, psychologist, or mental health counselor) in the previous 6 months and classified as 0 to 1 visit, 2 to 3 visits, 4 to 6 visits, and 7 or more visits. Satisfaction with diabetes care was based on the response to the question, “How would you rate your diabetes care overall?” (possible responses were excellent, good, fair, poor, and not applicable).

### Statistical Analysis

We used group-based trajectory modeling to identify trajectories of HbA_1c_ among youth with T1D using duration of diabetes (months) as the time scale via the PROC TRAJ macro of SAS statistical software version 9.4 (SAS Institute Inc), which fits a semiparametric (discrete mixture) model for longitudinal data using the maximum-likelihood method.^40,41,42,43,44^ Trajectory analysis uses all available data for a participant and is robust to data that are missing at random. Details about trajectory analysis have been described elsewhere.^43,44^ The optimal number of groups was determined based on Bayesian information criterion and having at least 5% of the sample in the smallest trajectory group. We named the trajectories based on the baseline HbA_1c_ value (from the initial research visit) and shape of the trajectory over the follow-up visits. We then calculated the posterior predicted probability for each participant of being a member of each trajectory group given his or her observed HbA_1c_ pattern. Participants were assigned to the trajectory group for which they had the greatest posterior probability for group membership. Multinomial regression was used to assess the association of race/ethnicity (non-Hispanic white vs non-Hispanic black vs Hispanic) with HbA_1c_ trajectory group membership. Youths who reported Asian or Pacific Islander, Native American, other, and unknown race/ethnicity (n = 34) were excluded from multinomial modeling. Non-Hispanic white was designated as the referent group.

All covariates were measured at baseline. Model 1 was unadjusted. Model 2 was adjusted for demographic factors (sex, age at diagnosis, and clinic site). Model 3 was additionally adjusted for clinical variables (body mass index *z* score, insulin regimen, insulin dose, and frequency of self-monitoring of blood glucose). Model 4 was further adjusted for socioeconomic position (highest parental education, household structure, and health insurance type).

Given previous findings of health inequity,^45^ we tested for sex- and age-related subgroups who may be particularly vulnerable to the effects of heath inequity. Modification of race/ethnicity effects by age and sex was tested by adding an interaction term (race/ethnicity × sex and race/ethnicity × age at diagnosis, respectively) to model 4. The nature of the modification was explored in models stratified by sex and the median age of diagnosis (9 years old). Because of limited sample size, for stratified analyses, race/ethnicity was categorized into non-Hispanic white and other (defined as non-Hispanic black, Hispanic, Asian or Pacific Islander, Native American, other, and unknown).

All analyses were completed in SAS software in 2017. Statistical significance was based on a 2-sided *P* value of .05. Descriptive analyses used the mean and standard deviation or median and interquartile range (IQR) for nonnormal distributions and for continuous variables and frequencies to describe categorical variables. The means and frequencies of demographic and clinical characteristics were compared using χ^2^ test for categorical variables and analysis of variance or Kruskal-Wallis test for continuous variables.

## Results

**Figure 2.**
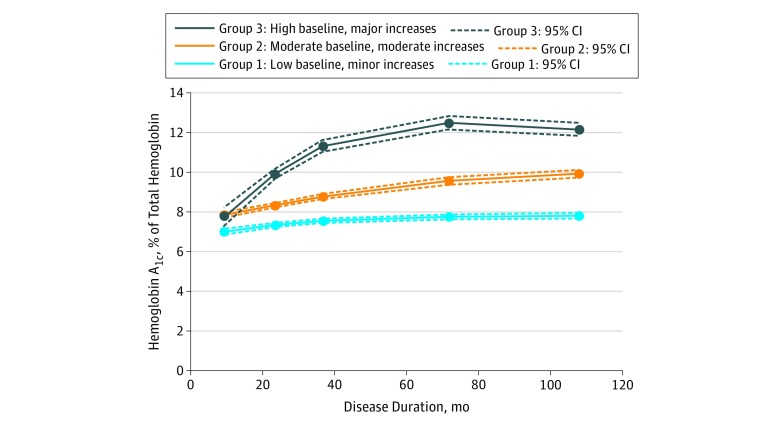
Trajectories of Hemoglobin A_1c_ in 1313 Patients With Type 1 Diabetes in the SEARCH for Diabetes in Youth Study Group-based trajectory modeling identified 3 distinct hemoglobin A_1c_ trajectories over a mean type 1 diabetes duration of 108 months. To convert hemoglobin A_1c_ to proportion of total hemoglobin, multiply by 0.01.

The sample of 1313 youths with T1D was 49.3% female (647 patients); 77.0% were non-Hispanic white (1011 patients); 10.7%, Hispanic (140 patients); 9.8%, non-Hispanic black (128 patients); and 2.6%, other race/ethnicity (34 patients) (Table 1). At the baseline visit, the mean (SD) age was 9.7 (4.3) years and the mean (SD) diabetes duration was 9.2 (6.3) months. Group-based trajectory modeling identified 3 distinct HbA_1c_ trajectories over a mean (SD) follow-up of 108 (16) months (9.0 [1.4] years) of diabetes duration: group 1, low baseline and mild increases (50.7% [666 patients]); group 2, moderate baseline and moderate increases (41.7% [548 patients]); and group 3, moderate baseline and major increases (7.5% [99 patients]) (Figure 2).

**Table 1.** Baseline Characteristics of 1313 Participants With Type 1 Diabetes by Hemoglobin A_1c_ Trajectory Group

Characteristic	No. (%)				*P* Value^a^
	Total Participants (N = 1313)	Group 1: Low Baseline and Mild Increases (n = 666)	Group 2: Moderate Baseline and Moderate Increases (n = 548)	Group 3: Moderate Baseline and Major Increases (n = 99)	
Age at diagnosis, mean (SD), y	8.9 (4.2)	8.8 (4.5)	8.5 (3.9)	11.3 (3.5)	<.001
Age at baseline, mean (SD), y	9.7 (4.3)	9.6 (4.5)	9.3 (3.9)	12.2 (3.5)	<.001
Diabetes duration, mean (SD), mo	9.2 (6.3)	9.0 (6.4)	9.3 (6.1)	10.4 (6.4)	.13
Female	647 (49.3)	316 (47.5)	280 (51.1)	51 (51.5)	.40
Nonwhite race/ethnicity^b^	302 (23.0)	102 (15.3)	153 (27.9)	47 (47.5)	<.001
Race/ethnicity^b^					
Non-Hispanic white	1011 (77.0)	564 (84.7)	395 (72.1)	52 (52.5)	<.001
Non-Hispanic black	128 (9.8)	34 (5.1)	69 (12.6)	25 (25.3)	
Hispanic	140 (10.7)	56 (8.4)	67 (12.2)	17 (17.2)	
Other	34 (2.6)	12 (1.8)	17 (3.1)	5 (5.1)	
Parental education					
Less than high school	48 (3.7)	20 (3.0)	20 (3.7)	8 (8.1)	<.001
High school graduate	180 (13.8)	61 (9.2)	93 (17.1)	26 (26.3)	
Some college (through associate’s degree)	441 (33.8)	184 (27.8)	219 (40.3)	38 (38.4)	
Bachelor’s degree or more	636 (48.7)	397 (60.0)	212 (39.0)	27 (27.3)	
Insurance					
None	19 (1.5)	8 (1.2)	8 (1.5)	3 (3.0)	<.001
Private	1052 (80.7)	586 (88.4)	402 (74.3)	64 (64.7)	
Medicaid	211 (16.2)	61 (9.2)	119 (22.0)	31 (31.3)	
Other	21 (1.6)	8 (1.2)	12 (2.2)	1 (1.1)	
Family structure					
Two-parent household	961 (73.6)	543 (81.9)	366 (67.4)	52 (52.5)	<.001
Single-parent household	311 (23.8)	109 (16.4)	161 (29.7)	41 (41.4)	
Other structure	33 (2.53)	11 (1.7)	16 (3.0)	6 (6.1)	
Insulin regimen					
Pump	106 (8.15)	67 (10.1)	36 (6.6)	3 (3.1)	.01
Long with short or rapid insulin, ≥3 times/d	418 (32.1)	225 (33.9)	164 (30.3)	29 (29.6)	
Long with other combination^c^	779 (59.8)	371 (56.0)	342 (63.1)	66 (67.4)	
Insulin dose, mean (SD), units/kg	0.63 (0.42)	0.59 (0.46)	0.66 (0.38)	0.73 (0.38)	.001
Blood glucose monitoring, times/d					
<1	10 (0.8)	14 (2.1)	11 (2.0)	4 (4.0)	<.001
1-3	148 (11.5)	64 (9.6)	58 (10.6)	26 (26.5)	
≥4	1134 (88.8)	588 (88.3)	478 (87.4)	68 (70.4)	
Body mass index *z* score, mean (SD)	0.58 (0.97)	0.40 (0.92)	0.66 (1.00)	0.68 (1.10)	.02
Diabetes care visits in past 6 mo, No.^d^					
Mean (SD)^d^	3.9 (2.9)	3.9 (2.9)	4.1 (3.0)	3.6 (2.6)	.31
0-1	173 (13.2)	95 (14.3)	65 (11.9)	13 (13.1)	.12
2-3	479 (36.5)	228 (34.2)	211 (38.5)	40 (40.4)	
4-5	383 (29.2)	211 (31.7)	142 (25.9)	30 (30.30)	
≥6	278 (21.2)	132 (19.8)	130 (23.7)	16 (16.2)	
Other care visits in past 6 mo, No.^d^					
Mean (SD)^d^	5.0 (4.1)	4.8 (3.9)	5.1 (4.3)	5.0 (4.5)	.44
0-1	175 (13.3)	83 (12.5)	73 (13.3)	19 (19.2)	.23
2-3	384 (29.3)	207 (31.1)	153 (27.9)	24 (24.2)	
4-6	424 (33.1)	225 (33.8)	182 (33.2)	27 (27.3)	
≥7	320 (24.4)	151 (22.7	140 (25.6)	29 (29.3)	
Satisfaction with diabetes care^e^					
Excellent	938 (72.4)	505 (77.2)	382 (70.6)	51 (54.8)	<.001
Good	288 (22.4)	127 (19.4)	133 (24.6)	28 (30.1)	
Fair	49 (3.8)	16 (2.5)	21 (3.9)	12 (12.9)	
Poor	5 (0.4)	1 (0.2)	3 (0.6)	1 (1.1)	

^a^ *P* values based on use of χ^2^ test and analysis of variance or Kruskal-Wallis test, as appropriate based on model assumptions.

^b^ Self-reported race and ethnicity were collected using 2000 US Census questions. White was defined as non-Hispanic white. Nonwhite was defined as non-Hispanic black, Hispanic, or other. Other was defined as Asian or Pacific Islander, Native American, other, or unknown.

^c^ Includes 2 or more times per day or any insulin combination (excluding long), 3 or more times per day or any insulin(s) taken once per day, or any insulin combination (excluding long) 2 or more times per day.

^d^ Diabetes care measured by frequency of visits with pediatric endocrinology, adult diabetologist, or nurse diabetes educator in the previous 6 months. Other care measured by frequency of visits with nondiabetes caregivers. Data are self-reported.

^e^ Based on response to the question, “How would you rate your diabetes care overall?” Possible answers were excellent, good, fair, poor, and not applicable.

The prevalence of black and Hispanic youth was the highest in group 3 and the lowest in group 1 (non-Hispanic black patients made up 5.1% of group 1, 12.6% of group 2, and 25.3% of group 3; Hispanic patients made up 8.4% of group 1, 12.2% of group 2, and 17.2% of group 3). For non-Hispanic black patients, the difference between group 1 and group 2 was 7.5% (95% CI, 4.2%-10.7%; *P* < .001); between group 1 and group 3, 20.2% (95% CI, 11.4%-28.9%; *P* < .001); and between group 2 and group 3, 12.6% (95% CI, 3.7%-21.7%; *P* = .001). For Hispanic patients, the difference between group 1 and group 2 was 3.8% (95% CI, 0.4%-7.3%; *P* = .03); between group 1 and group 3, 8.8% (95% CI, 1.0%-16.5%; *P* = .006); and between group 2 and group 3, 5.0% (95% CI, 3.0%-12.9%; *P* = .18). Group 3 was composed of 47.5% nonwhite youths (47 patients) (Table 1). Table 2 depicts the odds ratios (ORs) for non-Hispanic black and Hispanic vs non-Hispanic white race/ethnicity and HbA_1c_ trajectory group in a series of sequentially adjusted models. Non-Hispanic black youth had 7.98 higher odds than non-Hispanic white youth of being in the highest HbA_1c_ trajectory group relative to the lowest HbA_1c_ trajectory group (unadjusted OR of non-Hispanic black race in group 3 vs group 1, 7.98; 95% CI, 4.42-14.38). After adjustment for baseline demographic characteristics, clinical factors, and socioeconomic position, non-Hispanic black youth had 4.54 times higher odds than non-Hispanic white youth of being in the highest HbA_1c_ trajectory group relative to the lowest HbA_1c_ trajectory group (adjusted OR [aOR] of non-Hispanic black race in group 3 vs group 1, 4.54; 95% CI, 2.08-9.89). Hispanic youth had 3.29 higher unadjusted odds than non-Hispanic white youth of being in the highest HbA_1c_ trajectory group relative to the lowest HbA_1c_ trajectory group (unadjusted OR of Hispanic ethnicity in group 3 vs group 1, 3.29; 95% CI, 1.78-6.08). Adjustment for baseline demographic characteristics, clinical factors, and socioeconomic position did not fully attenuate the association (aOR of Hispanic ethnicity in group 3 vs group 1, 2.24; 95% CI, 1.02-4.92). Adjustment for clinical variables diminished statistical significance associated with the moderate HbA_1c_ trajectory (aOR of Hispanic ethnicity in group 2 vs group 1, 1.43; 95% CI, 0.90-2.27 vs unadjusted OR, 1.71; 95% CI, 1.17-2.49).

**Table 2.** Association of Black and Hispanic Race/Ethnicity, Compared With Non-Hispanic White Race/Ethnicity, With Hemoglobin A_1c_ Trajectory Groups in 1011 Patients

Model^a^	Odds Ratio (95% CI)					
	Black Race (n = 128)^b^			Hispanic Ethnicity (n = 140)^b^		
	Group 1: Low Baseline and Mild Increases	Group 2: Moderate Baseline and Moderate Increases	Group 3: Moderate Baseline and Major Increases	Group 1: Low Baseline and Mild Increases	Group 2: Moderate Baseline and Moderate Increases	Group 3: Moderate Baseline and Major Increases
Model 1	1 [Reference]	2.90 (1.88-4.46)	7.98 (4.42-14.38)	1 [Reference]	1.71 (1.17-2.49)	3.29 (1.78-6.08)
Model 2	1 [Reference]	3.00 (1.92-4.67)	9.94 (5.15-19.20)	1 [Reference]	1.67 (1.08-2.58)	3.56 (1.75-7.21)
Model 3	1 [Reference]	2.50 (1.54-4.05)	7.50 (3.68-15.26)	1 [Reference]	1.43 (0.90-2.27)	3.32 (1.60-6.91)
Model 4	1 [Reference]	1.73 (1.04-2.90)	4.54 (2.08-9.89)	1 [Reference]	1.16 (0.71-1.89)	2.24 (1.02-4.92)

^a^ Model 1 was unadjusted. Model 2 was adjusted for demographic characteristics (age at diagnosis and clinic site). Model 3 further adjusted for body mass index (calculated as weight in kilograms divided by height in meters squared) *z* score, insulin regimen, insulin dose, and frequency of blood glucose monitoring. Model 4 further adjusted for socioeconomic position (maximum parental education, household structure, and health insurance type).

^b^ Self-reported race and ethnicity were collected using 2000 US Census questions and categorized as non-Hispanic white, non-Hispanic black, Hispanic, and other (Asian, Native American, Pacific Islander, other, and unknown). Respondents who self-reported as other were excluded from these analyses due to small sample size (n = 34).

The association of race/ethnicity and HbA_1c_ trajectory was modified by sex (*P* for interaction = .04) (Table 3). Nonwhite male patients had significantly elevated odds of membership in the highest HbA_1c_ trajectory group (OR of group 3 vs group 1, 5.34; 95% CI, 2.16-13.2) and moderate HbA_1c_ trajectory group (OR of group 2 vs group 1, 2.06; 95% CI, 1.18-3.57) relative to non-Hispanic white male patients. The associations were not significant in female patients (aOR of group 3 vs group 1, 1.48; 95% CI, 0.65-3.39 and aOR of group 2 vs group 1, 1.00; 95% CI, 0.61-1.64). The association of race/ethnicity and HbA_1c_ trajectory was also modified by age at diagnosis (*P* for interaction = .02) (Table 3). Nonwhite youths diagnosed at or younger than 9 years had significantly elevated odds of membership in the highest HbA_1c_ trajectory group (aOR of group 3 vs group 1, 5.37; 95% CI, 1.91-15.1) and the moderate HbA_1c_ trajectory group (aOR of group 2 vs group 1, 2.04; 95% CI, 1.23-3.37). The association was not significant in youth who were diagnosed when they were older than 9 years (aOR of group 3 vs group 1, 1.65; 95% CI, 0.77-3.51 and aOR of group 2 vs group 1, 0.96; 95% CI, 0.55-1.65).

**Table 3.** Association of Nonwhite Race/Ethnicity, Compared With Non-Hispanic White Race/Ethnicity, With Hemoglobin A_1c_ Trajectory Group, Stratified by Sex and Age at Diagnosis^a^

Model^b^	Odds Ratio (95% CI)			*P* Value for Interaction
	Group 1: Low Baseline and Mild Increases	Group 2: Moderate Baseline and Moderate Increases	Group 3: Moderate Baseline and Major Increases	
Sex				
Female (n = 581)	1 [Reference]	1.00 (0.61-1.64)	1.48 (0.65-3.39)	.04
Male (n = 593)	1 [Reference]	2.06 (1.18-3.57)	5.34 (2.16-13.2)	
Age at diagnosis, y	1 [Reference]			
≤9 (n = 611)	1 [Reference]	2.04 (1.23-3.37)	5.37 (1.91-15.1)	.02
>9 (n = 564)	1 [Reference]	0.96 (0.55-1.65)	1.65 (0.77-3.51)	

^a^ Self-reported race and ethnicity were collected using 2000 US Census questions. White was defined as non-Hispanic white. Nonwhite was defined as non-Hispanic black, Hispanic, Asian or Pacific Islander, Native American, other, or unknown.

^b^ Fully adjusted for age at diagnosis, clinic site, maximum parental education, household structure, health insurance type, body mass index (calculated as weight in kilograms divided by height in meters squared) *z* score, insulin regimen, insulin dose, and frequency of blood glucose monitoring.

## Discussion

In a large, population-based multiethnic cohort of youth with T1D, we found 3 distinct HbA_1c_ trajectories that deteriorated over a mean (SD) follow-up of 9.0 (1.4) years (range, 6.1-13.3 years) following diabetes diagnosis, reinforcing that early youth and the transition to adulthood are high-risk periods for worsening glycemic control.^3,7,8^ Black race and Hispanic ethnicity were associated with membership in the highest and most rapidly increasing (worsening) HbA_1c_ trajectory group.

We tested the association of race/ethnicity with HbA_1c_ trajectory by adjusting for other variables, including clinical factors and socioeconomic position. For example, prescribing practices may vary based on race/ethnicity^27^ and insulin pump use is known to be higher in white youth than non-Hispanic black or Hispanic youth.^28^ Lower socioeconomic position has been proposed as a major mediator of the association of race/ethnicity with health outcomes,^29,30,31^ including T1D complications, due to poorer self-management among persons whose socioeconomic conditions are less favorable.^20,46^ Despite adjustment for these known risk factors, black race remained significantly associated with HbA_1c_ trajectory. Similarly, adjustment for demographic characteristics, clinical variables, and socioeconomic position did not fully attenuate the association of Hispanic ethnicity with the highest HbA_1c_ trajectory, where the OR remained significantly elevated, suggesting remaining impact of inequity in this group. Evidence of disparity in glycemic control trajectory that exists particularly among nonwhite male patients and nonwhite youth with diabetes diagnosis at an early age (≤9 years) is consistent with previously reported patterns in acute glycemic complications that are more common among the youngest patients and male patients of all ages.^47^

An important finding of the trajectory analysis was that the highest HbA_1c_ trajectory subgroup also showed the highest mean HbA_1c_ level at baseline, which occurred at a mean (SD) of 9.8 (6.3) months following diagnosis. This suggests that glycemic control obtained in the first year following diagnosis may confer information about longitudinal trends over time. Furthermore, the magnitude of racial/ethnic inequity over the longitudinal data are striking. Group 3 diverged over the follow-up period to give vastly different mean HbA_1c_ measures at the cohort visit that may translate to significant increases in the risk for complications of diabetes based on evidence from the Diabetes Control and Complications Trial (DCCT) and the Epidemiology of Diabetes Interventions and Complications Study (EDIC).^1,2,3,48^ Disparity in glycemic control across trajectory groups in the present analyses even exceeds differences reported across groups of the DCCT/EDIC trial (which compared a median HbA_1c_ of 7% of total hemoglobin in the intensive insulin treatment group with a median HbA_1c_ of 9% of total hemoglobin in the conventional group), suggesting that those risk estimates may be conservative for youth who additionally face a longer period of disease-related exposures.^49^

Previous studies have shown that the migration status of parents is associated with glycemic control among youth with T1D.^50^ To address potential differences, we examined a subset of the sample with data on parental nativity (ie, US born vs foreign born) and found no significant differences across HbA_1c_ trajectory groups. Adjustment for parental nativity did not attenuate the association of black race or Hispanic ethnicity with the moderate or highest HbA_1c_ trajectory group, although the analysis is limited by small sample size (data not shown). Differences in youth and parental nativity status likely warrant future study in adequately powered samples.

Given the complexity of the study of race and health outcomes in the United States, in which health risks associated with race/ethnicity are not inherent but instead may signal underlying inequalities,^51^ we posit that our results may reflect health inequity in T1D operating at multiple levels. The social determinants of health operating outside of the health care system, including aspects of the physical environment, food security, social integration, barriers to health care,^52^ and complex patterns in health care utilization,^53,54^ may create race-based groups of individuals for whom glycemic control is challenged by inconsistencies in the availability of resources or support for T1D management. In general, adverse childhood experiences among nonwhite youth have been shown to result in a myriad of psychological and medical sequelae later in life.^55^

There may also be modifiable aspects within the health care system, including racial/ethnic differences in the interpersonal dynamics of interactions between patients or parents and health care professionals that occur in pediatric clinical settings, extending from implicit bias and microaggressions to stereotyping, prejudice, and macroaggressions.^56^ Nonwhite youth and families report overtly weakened patient–health care professional communication and decreased participatory decision making.^32,33^ Implicit bias, the unconscious attitudes that unintentionally influence behavior, may affect health care professionals’ medical management decisions^56^ and perceptions about black, Hispanic, and young people of color in terms of disease experience^57^ and patient compliance.^58^ Higher levels of perceived bias or discrimination have been linked to worse diabetes care.^59,60^ The direct effect of implicit bias on HbA_1c_ has not been well studied in pediatric diabetes. Finally, while social stigma associated with T1D is known,^61,62,63^ it may be more pronounced in specific communities where health literacy and resources are lacking or where T1D is significantly less common than type 2 diabetes. Nonwhite youth may struggle with misunderstanding and stigma that act as chronic stressors that indirectly affect glycemic control via psychosocial or behavioral effects,^64,65^ resulting in impaired self-care strategies or maladaptive coping behaviors that damage health.^66^

### Limitations

A limitation of the study is that the observed inequity after adjustment for other factors may reflect racial and ethnic differences in the validity of HbA_1c_ as a measure of average glycemia owing to racial differences in the glycation of hemoglobin or other factors affecting red blood cell turnover.^67,68,69^ However, the between-race differences that have been reported are small (0.4 percentage point in HbA_1c_^69^) relative to the differences in the present study, where the mean (SD) HbA_1c_ of group 3 was 12.2% (1.5%) of total hemoglobin at the last visit, roughly 2.2% higher than group 2 and 4.4% higher than group 1 at that time. Combining individuals of many races, ethnicities, and cultures into single categories for analysis may result in residual confounding and underemphasize within-group heterogeneity. We are careful to avoid implying that all nonwhite youth have poor control; in our data, nearly a quarter of nonwhite youth had an HbA_1c_ at or below 7.4% of total hemoglobin at the cohort visit (data not shown). Several of the variables measured at baseline may change over time, including health insurance status. Adjustment variables may provide information for future work that will delve into what drives the inequities. For example, measures of socioeconomic position may be improved by including other measures such as the ability to pay for medication, heath literacy, housing security, or food security. We did not control for diet and physical activity in these analyses. A larger sample may identify additional trajectories that capture the experience of smaller subpopulations, such as individuals who initially have low HbA_1c_ that deteriorates later in the course of T1D. The outcome of trajectory group necessitated the use of logistic regression modeling, which may overestimate effect estimates, particularly when the outcome is common.^70,71^ Finally, there were relatively small numbers of participants across groups in the analyses stratified by sex and age at diagnosis. Larger studies are needed to further explore interactions and identify nonwhite youth who are at the highest risk for poor glycemic control over time. Finally, associations of data-driven trajectory models should be confirmed with future analyses that quantify and compare differences in longitudinal HbA_1c_ across racial/ethnic groups.

However, the study has several strengths, including the large, well-characterized, multiethnic cohort;^72^ the extended follow-up period; and the use of an analytic approach to characterize multiple common HbA_1c_ trajectories and understand associated individual characteristics from an extensive collection of covariates.

## Conclusions

Compared with non-Hispanic white youth with T1D, non-Hispanic black youth, Hispanic youth, and youth with other racial/ethnic backgrounds who are male and diagnosed earlier in life are more likely to show rapid deterioration in glycemic control within 9 years of T1D diagnosis. The findings of this study can be used to inform future research on the identification of factors that contribute to and reinforce racial and ethnic disparity among youth with T1D, particularly nonwhite male patients and nonwhite youth diagnosed earlier in life.
